# Supplementary material for: Estimating the effects of legalizing recreational cannabis on newly incident cannabis use
Source: PLoS One. 2022 Jul 21;17(7):e0271720. doi: 10.1371/journal.pone.0271720 (PMC9302774; doi:10.1371/journal.pone.0271720)
Supplement: S2 Fig — (PDF) [file pone.0271720.s002.pdf]

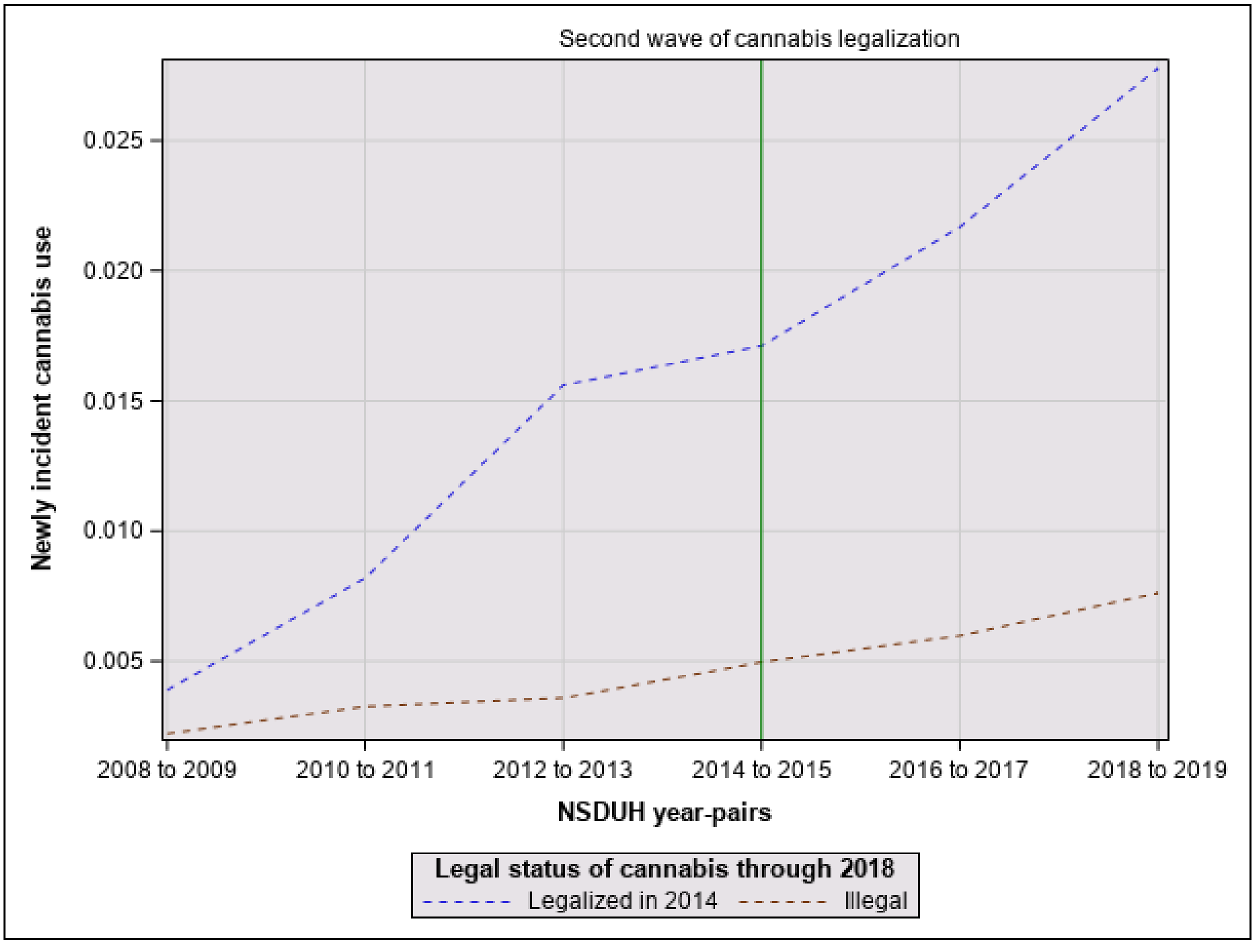

S2 Fig. Cannabis incidence in 21 and older age group, second wave legalizing states vs untreated states.
