# Supplementary figures and images for: Estimating the effects of legalizing recreational cannabis on newly incident cannabis use
Source: PLoS One. 2022 Jul 21;17(7):e0271720. doi: 10.1371/journal.pone.0271720 (PMC9302774; doi:10.1371/journal.pone.0271720)

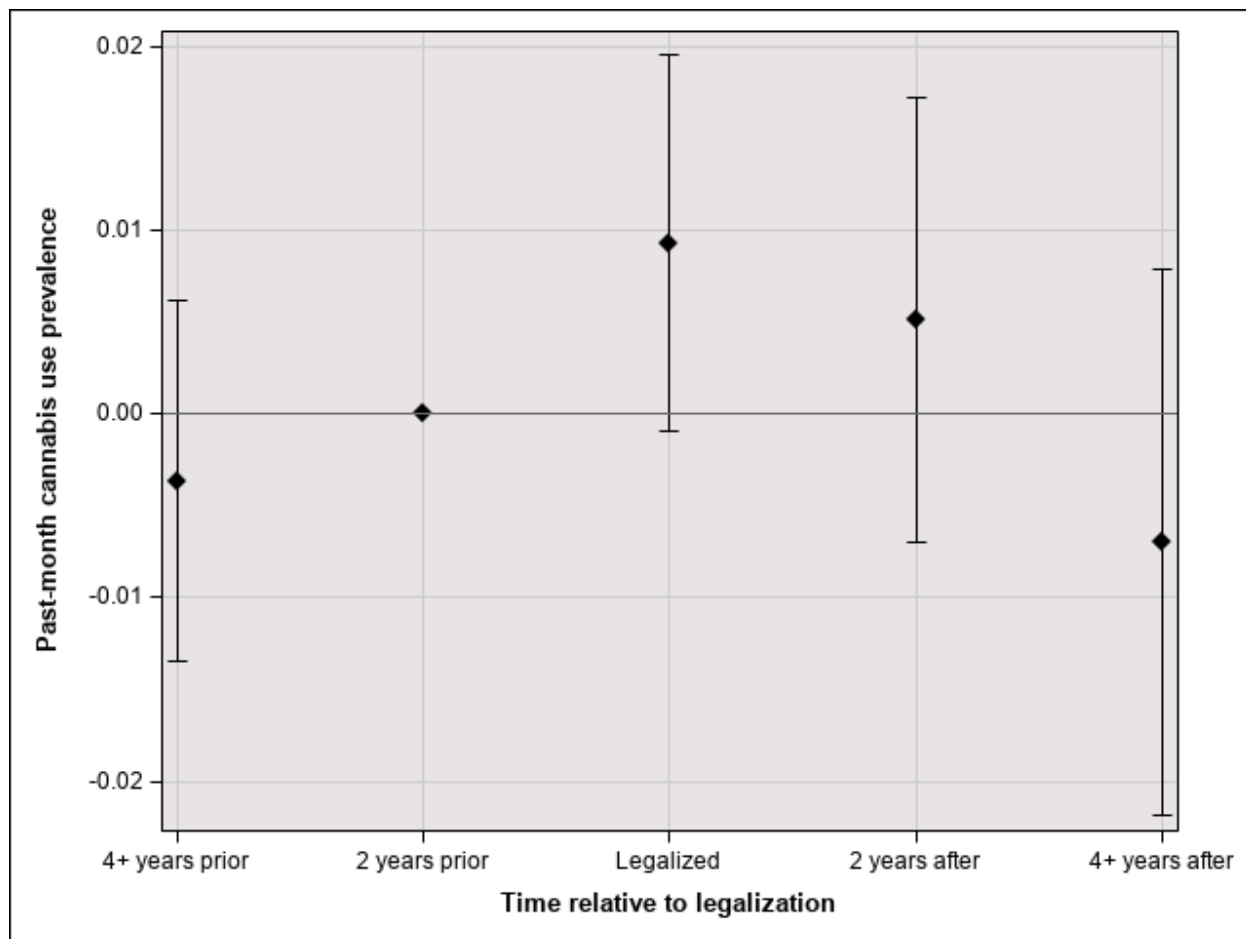

**S7 Fig. Effect of time since legalization on past-month cannabis prevalence in the 12-to-20-age-group.**

Supplement: S7 Fig — (PDF) [file pone.0271720.s007.pdf]
