# Supplementary material for: Estimating the effects of legalizing recreational cannabis on newly incident cannabis use
Source: PLoS One. 2022 Jul 21;17(7):e0271720. doi: 10.1371/journal.pone.0271720 (PMC9302774; doi:10.1371/journal.pone.0271720)
Supplement: S9 Fig — (PDF) [file pone.0271720.s009.pdf]

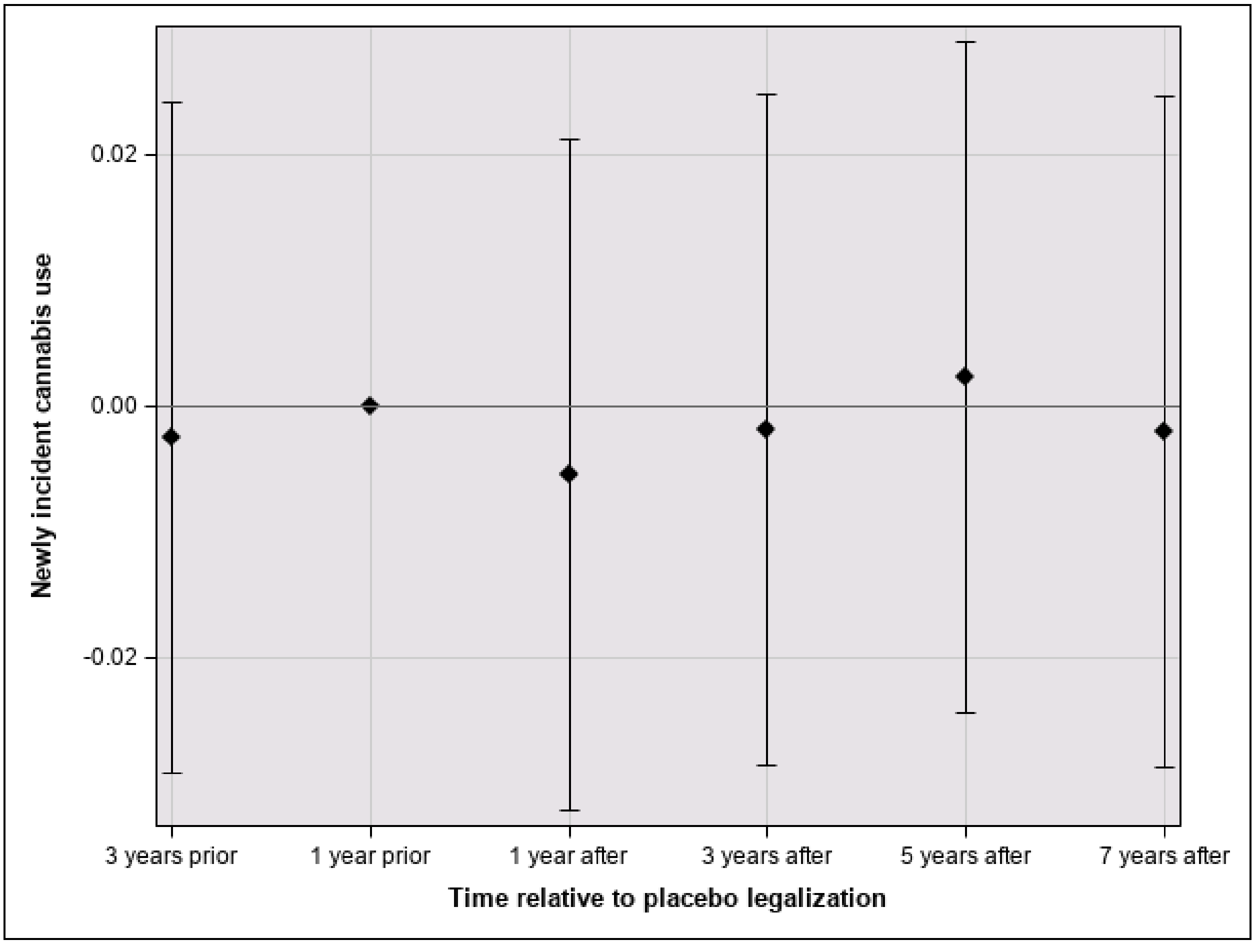

S9 Fig. Placebo effect of time since cannabis legalization on cannabis incidence in the 12-to-20-age-group.
