# Supplementary material for: Estimating the effects of legalizing recreational cannabis on newly incident cannabis use
Source: PLoS One. 2022 Jul 21;17(7):e0271720. doi: 10.1371/journal.pone.0271720 (PMC9302774; doi:10.1371/journal.pone.0271720)
Supplement: Attachment — Submitted filename: Response to Reviewers.docx [file pone.0271720.s010.docx]

| Reviewer and Comment # | Comment | Response |
| --- | --- | --- |
| Journal Requirement #1 | When submitting your revision, we need you to address these additional requirements.  Please ensure that your manuscript meets PLOS ONE's style requirements, including those for file naming. The PLOS ONE style templates can be found at  https://journals.plos.org/plosone/s/file?id=wjVg/PLOSOne_formatting_sample_main_body.pdf and  https://journals.plos.org/plosone/s/file?id=ba62/PLOSOne_formatting_sample_title_authors_affiliations.pdf | Thank you for providing these guiding documents on formatting. The manuscript and associated files have been formatted according to the journal’s requirements.  References have been changed to adhere to the Vancouver style |
| Journal Requirement #2 | Please provide additional details regarding participant consent. In the ethics statement in the Methods and online submission information, please ensure that you have specified what type you obtained (for instance, written or verbal, and if verbal, how it was documented and witnessed). If your study included minors, state whether you obtained consent from parents or guardians. If the need for consent was waived by the ethics committee, please include this information. | Thank you for this suggestion, I added the following sentence to the first paragraph on the methods study population: “As this research used publicly available and anonymized data, the research was determined as not human subjects research by the Michigan State University Institutional Review Board on 8/26/2021 (MSU Study ID: STUDY00006620)” |
| Journal Requirement #3 | Thank you for stating in your Funding Statement:  (There was no research support from the cannabis or other non-federal or non-university sources.  BWM, MHR, CEM, and JCA wish to acknowledge support from the Michigan State University Vice President for Graduate Studies and Research (university funds) as well as federal research grant support from the National Institutes of Health (5R25DA051249). BWM and MHR also wish to acknowledge the Michigan State University Graduate School for funding from the Graduate Enrichment Fellowship and the University Distinguished Fellowship, respectively.  The funders had no role in study design, data collection and analysis, decision to publish, or preparation of the manuscript.) Please provide an amended statement that declares *all* the funding or sources of support (whether external or internal to your organization) received during this study, as detailed online in our guide for authors at http://journals.plos.org/plosone/s/submit-now.  Please also include the statement “There was no additional external funding received for this study.” in your updated Funding Statement.  Please include your amended Funding Statement within your cover letter. We will change the online submission form on your behalf. | Added the sentence “There was no additional external funding received for this study.” in the updated Funding Statement and included it in the cover letter |
| Journal Requirement #4 | We noted in your submission details that a portion of your manuscript may have been presented or published elsewhere.  (PLOSM , presubmission inquiry,  PMEDICINE-D-22-00520) Please clarify whether this publication was peer-reviewed and formally published. If this work was previously peer-reviewed and published, in the cover letter please provide the reason that this work does not constitute dual publication and should be included in the current manuscript. | The work was not peer-reviewed. It was published online in the medrxiv pre-print server: doi: https://doi.org/10.1101/2022.01.26.22269900 |
| Journal Requirement #5 | Please include your full ethics statement in the ‘Methods’ section of your manuscript file. In your statement, please include the full name of the IRB or ethics committee who approved or waived your study, as well as whether or not you obtained informed written or verbal consent. If consent was waived for your study, please include this information in your statement as well. | The following sentence was added to the first paragraph of the methods section: “As this research used publicly available and anonymized data, the research was determined as not human subjects research by the Michigan State University Institutional Review Board on 8/26/2021 (MSU Study ID: STUDY00006620)” |
| Journal Requirement #6 | Please include your tables as part of your main manuscript and remove the individual files. Please note that supplementary tables (should remain/ be uploaded) as separate "supporting information" files" | Thank you for the suggestion, table 1 is included in the main manuscript on page 12. |
| Journal Requirement #7 | Please include captions for your Supporting Information files at the end of your manuscript, and update any in-text citations to match accordingly. Please see our Supporting Information guidelines for more information: http://journals.plos.org/plosone/s/supporting-information. | Added the required page for Supporting information with corrected supplemental figure titles. Updated in-text citations to match. |
| Reviewer 1 #1 | None | We thank reviewer one for a succinct and accurate summary of our work. |
| Reviewer 2 #1 | could it not be that no increase is being seen among those for whom use remains illegal simply due to that very fact and their mistrust of reporting illegal behavior? | We thank the reviewer for bringing up this important limitation. We discussed this issue as an extension of the limitations of self-report in our section on the limitations of this research. To reiterate: this is absolutely a possibility that we acknowledge, but it has yet to be shown empirically. However, past research has shown that cannabis is one of the most reliably and valid questions in these surveys and the standardized audio computer-assisted self-interview modules were designed to reduce biases of this type. We did add sub-section headings in our discussion section to more clearly emphasize where the limitations of this study are discussed. |
| Reviewer 2 #2 | As a side note, cannabis policy is not made at the “county” level in many states, perhaps excepting WA and CO, but at sub-county (town, village, other type of municipality level) | Thank you for this note. Our reading of the policy literature on this topic shows that states (as you reference) differ in terms of the level of government which is allowed jurisdiction over the issue. In another reading, we do see that you are correct that we do seem to use the term county as a stand in for both counties and municipalities. We have changed some of the language in the manuscript to be more inclusive of the other levels of government that regulate this policy. |
| Reviewer 2 #3 | The conclusion that there is no increase in cannabis incidence among youth seem also to conflict with prior findings of several studies, so I would be curious how the authors reconcile these findings with those. | To be clear, no publication to date has studied the incidence of new cannabis use. Therefore, there cannot be a conflict in findings regarding incidence. We did show no increase in past 30 day prevalence among those aged 12-20. However, in our reading of the literature, studies that showed an increase in cannabis use prevalence among youth were in the minority and tended to be earlier papers, some of which used the date of legal change instead of the date of the effective change (i.e., Cerda et al 2017 and Paschall, García-Ramírez, & Grube, 2021). The prevalence of cannabis use disorder among youth increased in both studies that used that outcome, but that is a very different outcome. |
| Reviewer 2 #4 | On a more technical note, the way that incident cannabis use was defined using the NSDUH variables was not clear to me. I read it several times. Maybe including the specific variables used, just making it more explicit, would be helpful for readers. | Thank you for this helpful suggestion, we have added the NSDUH variables required for this analysis. |
| Reviewer 2 #5 | Would it potentially be possible to model both incidence and past 30-day use outcomes using this alternative method (which I found compelling) to examine whether results are consistent or whether they diverge. | We thank the reviewer for this suggestion. We did in fact model both incidence as well as past 30 day use. The past 30 day use outcomes was used as a robustness check in the way the reviewer suggested. However, the results are somewhat tucked-away in that section of the results and included in the supplemental figures S6 and S7. |
| Reviewer 2 #6 | I am not clear on what the authors mean by allowing for ‘heterogeneity’ that should be considered with RCL effects in this context? Among various groups or only over time? I believe it is clear there is a policy lag not only because that is natural, but because there is often a lengthy delay between “passage/adoption” of a RCL which then leads to more immediate “decriminalization” prior to dispensary openings. | We thank the reviewer for this helpful clarification. In most cases in this paper, we were referring to the heterogeneity over time as suggested by the reviewer (i.e., policy lag). We modified some of the language in the paper to make this reference clearer. |
| Reviewer 2 #7 | I’ve included several references that seem relevant with the hope that they may be helpful. The conclusions cited regarding whether there have been increases in use among 12-17 year olds (or those under age 21) overall and/or by RCL status seem not as consistent as suggested, potentially. | We that the reviewer for these suggested articles and have added the two that were relevant to recreational cannabis policy associations and effects to our summary of the literature. |
